# The Peripheral Cannabinoid Receptor Type 1 (CB_1_) as a Molecular Target for Modulating Body Weight in Man

**DOI:** 10.3390/molecules26206178

**Published:** 2021-10-13

**Authors:** Saoirse Elizabeth O’Sullivan, Andrew S. Yates, Richard K. Porter

**Affiliations:** 1 Artelo Biosciences, Solana Beach, CA 92075, USA; andy@artelobio.com; 2 School of Biochemistry & Immunology, Trinity Biomedical Sciences Institute (TBSI), Trinity College Dublin, D02R590 Dublin, Ireland; rkporter@tcd.ie

**Keywords:** CB_1_ receptor, peripheral, body weight, appetite, drug discover, cannabinoid

## Abstract

The cannabinoid 1 (CB_1_) receptor regulates appetite and body weight; however, unwanted central side effects of both agonists (in wasting disorders) or antagonists (in obesity and diabetes) have limited their therapeutic utility. At the peripheral level, CB_1_ receptor activation impacts the energy balance of mammals in a number of different ways: inhibiting satiety and emesis, increasing food intake, altering adipokine and satiety hormone levels, altering taste sensation, decreasing lipolysis (fat break down), and increasing lipogenesis (fat generation). The CB_1_ receptor also plays an important role in the gut–brain axis control of appetite and satiety. The combined effect of peripheral CB_1_ activation is to promote appetite, energy storage, and energy preservation (and the opposite is true for CB_1_ antagonists). Therefore, the next generation of CB_1_ receptor medicines (agonists and antagonists, and indirect modulators of the endocannabinoid system) have been peripherally restricted to mitigate these issues, and some of these are already in clinical stage development. These compounds also have demonstrated potential in other conditions such as alcoholic steatohepatitis and diabetic nephropathy (peripherally restricted CB_1_ antagonists) and pain conditions (peripherally restricted CB_1_ agonists and FAAH inhibitors). This review will discuss the mechanisms by which peripheral CB_1_ receptors regulate body weight, and the therapeutic utility of peripherally restricted drugs in the management of body weight and beyond.

## 1. Introduction

A well characterized feature of cannabis use is the stimulation of appetite and suppression of nausea. This effect of cannabis was thought to be primarily mediated by the phytocannabinoid Δ^9^-tetrahydrocannabinol (THC) binding to the CB_1_ receptor in key areas of the brain that regulate feeding and nausea including the hypothalamus (feeding), dorsal vagal complex and insular cortex (nausea), and nucleus accumbens and limbic areas (reward and motivation aspects of feeding) [1,2]. For this reason, cannabis has been used to treat the loss of appetite and body weight in several disorders. Synthetic forms of THC (dronabinol and Nabilone^®^) are approved for chemotherapy-induced nausea and vomiting across many countries, supported by meta-analyses of trial data in cancer patients, showing cannabinoids are effective at treating nausea and vomiting [3] and increasing appetite [4]. Dronabinol also causes significant weight gain in patients who are HIV-positive [5,6] (and is approved for HIV/AIDS-induced anorexia in some regions), young anorexic women [7,8], and in patients with Alzheimer’s disease [9].

Conversely, antagonising the CB_1_ receptor suppresses appetite and causes weight loss, and this has also been exploited therapeutically. The CB_1_ receptor blood–brain barrier (BBB) penetrable antagonist (and potentially inverse agonist [10]) Rimonabant (Acomplia^®^) was developed by Sanofi and licensed as an anti-obesity drug. Multiple randomized controlled trials (RCTs) showed that 20 mg rimonabant led to significant reductions in body weight and haemoglobin A1c (HbA1c), improved lipid profiles, and increased adiponectin (a metabolism-regulating adipokine) [11,12].

However, activation of central CB_1_ receptors can be associated with a side effect profile (such as euphoria, dizziness, memory loss, tiredness, and paranoia) that is not always well tolerated by patients, which has limited the use of centrally acting CB_1_ agonists in wasting disorders. Additionally, THC targets multiple receptors and ion channels other than cannabinoid receptors, some of which have weight loss promoting effects, such as GPR119 and PPARα. The currently licensed medicines in this space are dronabinol and Nabilone, both synthetic versions of THC, and their pharmacology may not be selective enough to achieve the desired weight gain in patients.

Antagonising central CB_1_ receptors is also associated with CNS-mediated neuropsychiatric side effects such as low mood, reduced joy, anxiety, depression, and suicidal ideology, due to the important role that CB_1_ receptors play in the brain’s reward system [13]. Indeed, rimonabant was withdrawn from clinical use in 2009 because of significant psychiatric adverse events (AEs) [11,14].

Considering the major contribution of the peripheral CB_1_ receptors in body weight control (for reviews see [1,15,16]), an alternative pharmaceutical development pathway is to peripherally restrict CB_1_ molecules to get the benefit of modulating peripheral CB_1_ receptors without the side effects of modulating central CB_1_ receptors. Such a strategy is being pursued by multiple pharmaceutical companies for the development of second- and third-generation anti-obesity CB_1_ receptor antagonists (for reviews see [10,17,18]). Peripherally restricted CB_1_ agonists are now also being used to gain the benefits of increased feeding and weight gain/maintenance in cancer cachexia. 

This review will discuss the mechanisms by which peripheral CB_1_ receptors regulate body weight, and the therapeutic utility of peripherally restricted drugs (both agonists, antagonists, and endocannabinoids modulators) in the management of body weight, and in novel therapeutic areas such as chronic kidney disease, pulmonary fibrogenesis, pain, and bladder disorders. The role of CB_1_ receptor signaling in the central control of feeding have been reviewed elsewhere [1,19].

## 2. Peripheral CB_1_ Receptors

**Figure 1:**
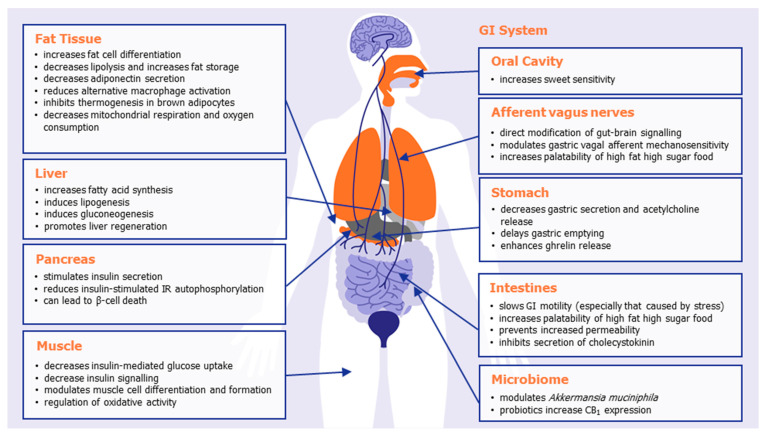
The effects of peripheral CB_1_ activation in promoting appetite, food storage, and weight gain.

At the peripheral level, extensive research has shown that CB_1_ receptor activation impacts the overall energy balance of mammals in a number of different ways, inhibiting satiety and emesis, increasing food intake, altering adipokine and satiety hormone levels, altering taste sensation, decreasing lipolysis, and increasing lipogenesis. Table 1 summarizes some of the known effects of CB_1_ activation in the various organs and body systems that play a role in body weight regulation, illustrated in Figure 1. The combined effect of peripheral CB_1_ activation is to promote appetite and promote energy storage and preservation, ultimately leading to weight gain or weight maintenance.

Important locations of peripheral CB_1_ receptors include the oral cavity, gastrointestinal tract, afferent vagus nerves, adipose tissue, liver, and pancreas. Mendizabal-Zubiaga and colleagues demonstrated CB_1_ to also be associated with mitochondria in skeletal, myocardial, and striated muscle, implicating CB_1_ with direct involvement in peripheral energy metabolism [20]. Selective knockdown of CB_1_ in adipose tissue [21], the liver [22], or skeletal muscle [23] all prevent diet-induced obesity or hyperphagia. Mice in whom CB_1_ was selectively knocked down in the intestinal epithelium did not have the preference for a Western style diet (with reduced caloric intake and meal size) normally observed in wild-type mice [24]. In a preclinical model of cachexia, it was recently shown that the potent, selective CB_1_/CB_2_ agonist WIN55,212-2 led to a significant reduction in the cachexia index and significantly prevented the cachexia-induced increase in gastric emptying [25].

There is strong correlative evidence from human studies that an active endocannabinoid system (ECS) is associated with visceral and subcutaneous fat accumulation [26], which is supported by many studies that CB_1_ activation promotes fat cell differentiation and fat storage (see Table 1 and Figure 1 for details). For instance, in a human study by Côté and colleagues, plasma 2-arachidonoylglycerol levels correlate positively with body mass index (BMI), waist girth, intra-abdominal adiposity, fasting plasma triglyceride, and insulin levels but negatively with high-density lipoprotein cholesterol and adiponectin [27]. However, visceral fat accumulation is an important correlate with insulin resistance, and higher circulating endocannabinoids have been associated with insulin resistant obese patients [28]. The fact that there are abundant CB_1_ receptors in visceral adipose tissue serves as means to target obesity and insulin resistance in human with peripheral CB_1_ receptor antagonists or indeed promote weight gain with peripheral CB_1_ receptor agonists. 

Activation of hepatic CB_1_ has been shown to be associated with obesity and insulin resistance (see Table 1). Measured observations include impaired metabolic function, impaired glucose and lipid metabolism, and augmentation of oxidative stress and inflammatory responses. Blocking peripheral CB_1_ in liver not only has weight loss potential, but also the potential to increase insulin sensitivity and glucose metabolism in humans while reducing the potential for hepatic steatosis [29]. It is worth noting that medicines that activate the CB_1_ receptor like nabilone may cause mild increase in serum liver enzymes but no cases of clinically apparent liver injury attributable to nabilone [30].

In human skeletal muscle studies, Eckardt and colleagues demonstrated that activation of the CB_1_ receptor decreases insulin-mediated glucose uptake and AKT activation in cultured cells [31]. Cavuoto and colleagues also demonstrated an attenuating effect of cannabinoid signalling on cultured human muscle cell oxidative pathways in vitro, while CB_1_ receptor antagonism increases whole body oxygen consumption [32]. In myotubes cultured from lean individuals, anandamide (AEA) treatment increases expression of pyruvate dehydrogenase kinase 4 (PDK4), an inhibitor of the pyruvate dehydrogenase complex, an enzyme which links glycolysis to the Krebs cycle, while CB_1_ antagonism decreases PDK4 expression. PDK4 is a negative regulator of glucose oxidative metabolism in mitochondria, but is an enzyme that is also physiologically inhibited to facilitate fatty acid oxidation. A series of studies from Iannotti and colleagues show an important role of the CB_1_ receptor in skeletal muscle cell differentiation and found that CB_1_ receptor antagonism (using rimonabant, intra peritoneally) was beneficial at preventing the locomotor deficits in an animal model of Duchenne muscular dystrophy [33,34]. Genetic inhibition of skeletal muscle receptor was also found to improve mitochondrial performance, whole-body muscle energy expenditure, and physical endurance [23]. These studies indicate an important role for CB_1_ in skeletal muscle function and metabolism.

Together, these data demonstrate that there are important direct effects of CB_1_ receptor activation in adipose tissue, the GI tract, skeletal muscle, and the liver that drive the effects of CB_1_ (agonism or antagonism) on body weight modulation.

**Table 1:** An overview of some of the effects of CB_1_ activation in various organs and body systems that play a role in metabolism. The combined effect of peripheral CB_1_ activation is to promote appetite, and energy storage and preservation.

System/Organ	Tissue/Cell	Effect of CB_1_ Activation
GI system	Oral cavity	CB_1_ receptors are expressed in type II taste cells that also express the sweet-taste receptor, and their activation increases sweet sensitivity [35]. CB_1_ receptors on the tongue increase gustatory nerve responses [35].
	Stomach	CB_1_ is expressed on acid-secreting parietal cells [36]. CB_1_ activation decreases gastric secretion and acetylcholine release [37]. CB_1_ activation delays gastric emptying [38]. CB_1_ is expressed in ghrelin-positive gastric mucosal cells [39]. CB_1_ activation enhances ghrelin release from the stomach [40].
	I cells of the small intestine	CB_1_ is expressed in enteroendocrine cells [41]. CB_1_ inhibits the secretion of the satiation hormone cholecystokinin [41].
	Intestines	CB_1_ activation slows GI motility, particularly stress-induced motility [42,43]. CB_1_ activation prevents increased intestine permeability (leaky guts) [44]. Intestinal CB_1_ activation important for palatability of high fat high sugar foods [45]. CB_1_ deletion in intestinal epithelium reduces western diet preferences [24].
	Afferent vagus nerves	CB_1_ receptors are expressed on vagal terminals [46,47]. Fasting increases CB_1_ expression on vagal afferent neurons [47]. The induction of feeding by peripherally CB_1_ activation is inhibited by vagal ablation [48]. CB_1_ activation modulates gastric vagal afferent mechanosensitivity to stretch/distension (leading to feeling of fullness) [39].
	Microbiome	CB_1_ receptor antagonism [49] or THC [50] increases Akkermansia muciniphila. Probiotic treatment increases CB_1_ and/or CB_2_ expression [51,52].
Fat tissue	Adipocytes	CB_1_ is expressed on adipocytes [53]. CB_1_ deletion protects adult mice from diet-induced obesity [21]. CB_1_ increases adipocyte differentiation and adipogenesis [54]. CB_1_ activation increases PPARγ expression, a major regulator of adipose function [52]. CB_1_ enhances fat storage and reduces lipolysis [54,55]. CB_1_ decreases adiponectin production [54,56]. CB_1_ reduces alternative macrophage activation [21].
	White adipocyte mitochondria	CB_1_ activation decreases mitochondrial respiration and oxygen consumption [57,58].
	Brown adipose tissue (BAT)	CB_1_ is upregulated during activation of BAT [59,60]. CB_1_ antagonism increases expression of uncoupling protein 1 (UCP-1) [61].
Liver	Hepatocytes	CB_1_ activation increases lipogenesis [62] CB_1_ activation increases fatty acid synthesis [62]. CB_1_ activation induces gluconeogenesis [63]. CB_1_ activation promotes liver regeneration by increasing mitotic progression [64]. CB_1_ knock-out mice are protected against diet-induced lipogenesis and steatosis [65].
Pancreas	Pancreatic β-cells	CB_1_ activation stimulates basal and glucose-dependent insulin secretion [66,67]. CB_1_ activation impedes insulin-stimulated IR autophosphorylation [68]. CB_1_ receptors can lead to β-cell death [69].
Muscle	Skeletal muscle cells	CB_1_ expression increases during skeletal muscle cell differentiation [31,33]. CB_1_ activation decreases insulin-mediated glucose uptake [31]. CB_1_ knockdown improves mitochondrial performance, increases whole-body muscle energy expenditure, and improves physical endurance [23]. CB_1_ receptor knockdown prevents diet-induced and age-induced insulin resistance [23].
	Myotubules	CB_1_ activation prevents myotubule formation [33]. CB_1_ activation inhibits sarcoplasmic Ca^2+^ release [70].
	Skeletal muscle satellite cells	CB_1_ activation inhibits satellite cell differentiation [34].
	Muscle Mitochondria	CB_1_ receptors regulates mitochondrial oxidative activity [20].

### 2.1. Effects of Peripheral CB_1_ Receptors on Appetite Hormones

In addition to the direct effects of CB_1_ activation in peripheral tissues, there are humoral and neuronal links between peripheral CB_1_ receptors and the central pathways controlling body weight through the modulation of key hormones that influence appetite.

Leptin is an adipose-derived hormone that acts on central receptors to reduce feeding and appetite, and leptin resistance is a feature of obesity. Cross-talk between central leptin and CB_1_ receptors has been well documented, but leptin resistance in diet-induced obese mice can be reversed by the peripherally restricted CB_1_ antagonist JD5037 [71], demonstrating that CB_1_ receptors also modulate leptin sensitivity at a peripheral level, and this plays an important role in the ability of peripheral CB_1_ blockade to mediate hypophagia and weight loss.

Ghrelin is a peptide hormone released in the gastrointestinal tract (mainly in the stomach and pancreas) and the brain that acts on receptors located on the vagus to stimulate appetite. The CB_1_ receptor is expressed in the neuroendocrine cells of the stomach that secrete ghrelin, and CB_1_ antagonism reduces ghrelin secretion, preventing appetite stimulation [40]. The peripheral-restricted CB_1_ antagonist LH-21 was also found to block ghrelin-induced hyperphagia in free feeding animals [72]. Thus, the anorexigenic effect of CB_1_ antagonists is at least partially a consequence of decreased gastric ghrelin secretion, and conversely CB_1_ activation in the stomach will increase ghrelin, stimulating appetite and food intake through ghrelin’s actions on the vagal nerve. This is supported by recent human studies that showed increased plasma levels of ghrelin after oral THC [73,74]. The ghrelin agonist anamorelin (Adlumiz^®^) has been approved in Japan for the treatment of cancer cachexia, demonstrating the utility of increasing ghrelin to improve anorexic and cachexic conditions [75]. 

Cholecystokinin (CCK) is a peptide hormone release from the duodenum during digestion, which acts as a hunger suppressant at receptors located on the vagus (mainly) and in the brain. The CB_1_ receptor is expressed on endocrine cells of the intestinal epithelium that secrete CCK, and activation of CB_1_ blocks the secretion of CCK (and the opposite true of CB_1_ antagonists) [41]. The same study showed that the hypophagic effect of a peripherally restricted CB_1_ antagonist in obese mice was reversed by co-administration with a CCK receptor antagonist, indicating the importance of CB_1_ regulation over this appetite suppressant hormone.

Together, these studies show that peripheral activation of CB_1_ modulates the activity of the key appetite-regulating hormones leptin, ghrelin, and CCK, whose receptors are located in the brain, or on the vagus nerve with direct influence on the brain via the gut–brain axis.

### 2.2. Gut–Brain Axis

In addition to the hormonal influence on the central integration of appetite, CB_1_ receptors are expressed on vagal terminals throughout the GI tract, playing a direct role in the modulation of afferent information to the brain and the regulation of food intake (see [76] for an extensive review on this topic). GI vagal afferents play an important role in the peripheral regulation of food intake via signalling the degree of distension of the stomach, which leads to feelings of fullness and satiety. CB_1_ activation inhibits the vagal afferent response to tension, thus preventing the feeling of fullness and allowing food consumption to continue [39,77].

Levels of the endogenous CB_1_ agonists anandamide and 2-AG increase in the intestine in the starved state or by (lipid) feeding, and this stimulates feeding, which is abolished after sensory deafferentation or CB_1_ receptor antagonism [48,78]. Argueta and DiPatrizio showed that the hyperphagia in mice given free access to a high-fat and sucrose diet was inhibited by a peripherally restricted CB_1_ antagonist [45]. These researchers went on to show that mice in whom CB_1_ was selectively knocked down in the intestinal epithelium did not have the preference for the high-fat and sucrose diet [24]. Thus, endogenous activation of CB_1_ in the intestine increases the palatability of food through gut–brain communication.

### 2.3. Microbiome

A novel mechanism of action for CB_1_ in the modulation of metabolism and body weight may be through modifications in the microbiome (see [79] for a recent review). Mehrpouya-Bahrami and colleagues found that a CB_1_ antagonist caused changes in the gut microbial community with an increase in Akkermansia muciniphila (Verrucomicrobiaceae family) and a decrease in the Lanchnospiraceae and Erysipelotrichaceae families, although it is not clear if this was a direct effect or secondary to the improvements in metabolic dysfunction [49]. Chronic THC treatment prevented the diet-induced obesity changes in gut microbiota, particularly causing an increase in Akkermansia muciniphila [50]. Probiotic treatment has also been shown to increase CB_1_ and CB_2_ expression in colonic mucosa and adipose tissue [52], which was associated with improvements in disease activity in dogs with gut dysmotility disturbances [51]. Conversely, studies using germ-free mice have shown that there is an upregulation of CB_1_ in the intestines that is reversed after faecal microbiota transfer [80]. These emerging studies suggest a link between the endocannabinoid system and gut bacteria that may play a role in the modulation of body weight by CB_1_ at the peripheral level.

## 3. Therapeutic Utility of Peripheral CB_1_ Receptors as Molecular Targets

### 3.1. Peripherally Restricted CB_1_ Antagonists

After the withdrawal of Rimonabant, researchers began developing peripherally restricted CB_1_ antagonists in obesity and diabetes. Molecules such as URB447 (a mixed CB_1_/CB_2_ neutral antagonist) [81], AM6545 (a CB_1_ neutral antagonist) [82], TXX-522 (a CB_1_ selective antagonist) [83], and LH-21 (a CB_1_ neutral antagonist) [72,84] were shown to reduce feeding and body weight gain in rodents. In models of diabetes, peripherally restricted CB_1_ antagonists improve glucose tolerance and insulin sensitivity [85]. This class of drugs also ameliorate other conditions associated with obesity and diabetes such as leptin resistance, fatty liver, and dyslipidemia [86,87] and reverse hyperphagia, body weight, and metabolic syndrome in a genetic model of Prader–Willi syndrome [88]. 

Another strategy to avoid the side effects of CB_1_ antagonists is through allosteric modulation of the CB_1_ receptor. The negative allosteric modulators ORG27569 [89], RVD-hemopressin(α) [90], and PSNCBAM-1 [91] reduce food intake with or without a reduction in body weight in rats.

In addition to metabolic disorders, preclinical research suggests peripherally restricted antagonists have beneficial effects on kidney diseases [92,93], liver fibrosis and steatosis [94,95,96], pulmonary fibrosis [97,98], and alcoholism [99] (see Table 2). In some cases, some third-generation compounds have been designed to inhibit more than one molecular target. For example, hybrid inhibitors of the CB_1_ receptor and inducible nitric oxide synthase (iNOS) show benefits in alcohol-drinking behaviors [100], kidney diseases [101], liver fibrosis [102], and skin fibrosis [103].

Several pharmaceutical companies are developing medicines to inhibit the peripheral CB_1_ receptor (see Table 2). 

Inversago Pharma has been granted rare paediatric disease designation by Food and Drug Administration (FDA) for the treatment of Prader–Willi syndrome with their peripherally restricted CB_1_ inverse agonist INV-101. The safety, tolerability, and pharmacokinetics of single ascending oral doses of INV-101 is being tested in healthy volunteers, although this trial is not recruiting at the time of writing (ClinicalTrials.gov Identifier: NCT04531150).

GFB-024 is a peripherally restricted CB_1_ inverse agonist monoclonal antibody intended to treat patients with severe insulin-resistant diabetic nephropathy (DN) in development by Goldfinch Bio (https://www.goldfinchbio.com/pipeline/gfb-024/ (accessed on 10 September 2021)). Goldfinch Bio have just announced a phase 1 clinical trial to evaluate the safety and pharmacokinetics of single and repeated dosing of GFB-024 in overweight healthy volunteers (ClinicalTrials.gov Identifier: NCT04880291). 

A phase 1 trial with the peripherally selective neutral CB_1_ antagonist TM38837 from 7TM Pharma has been conducted in healthy subjects [104], although it is unclear whether this is an active drug development program. 

JD5037 is a peripherally restricted CB_1_ inverse agonist developed by Jenrin Discovery and licensed to Corbus Pharmaceuticals (now CRB-4001), which is due to begin phase 1 testing in the first half of 2022 (https://www.corbuspharma.com/our-pipeline/endocannabinoid-system (accessed on 10 September 2021)).

**Table 2:** Potential therapeutic utility of peripherally restricted compounds targeting the CB_1_ receptor directly or indirectly.

	Peripherally Restricted CB_1_ Antagonists	Peripherally Restricted CB_1_ Agonists	Peripherally Restricted FAAH Inhibitors
Preclinical research	Obesity [81,82,83,86] Type 2 diabetes [85,105] Prader–Willi syndrome [88] Chronic kidney disease [101] Diabetic nephropathy [93] Alcoholic liver steatosis [94] Alcoholism [99,100] Non-alcoholic liver steatosis [96] Obesity-related liver steatosis [95] Liver fibrosis [102] Pulmonary fibrogenesis [97,98] Skin fibrosis [103]	Inflammatory pain [106,107] Neuropathic pain [107,108] Bone cancer pain [109] Chemotherapy-induced pain [110] Migraine and medication overuse headache [111] Spasticity in multiple sclerosis [112] Gastrointestinal motility in colitis [42,43] Anticipatory nausea [113] Cardiac disease [114]	Neuropathic pain [115] Chemotherapy-induced neuropathy [116] Inflammatory pain [115,117,118] Diabetic neuropathy [119] Visceral pain [115] Migraine [120,121] Anticipatory nausea [113] Cystitis [122] Bladder overactivity [123] Gastric lesions [118]
Clinical research	INV-101 in Prader–Willi syndrome (PWS) and non-alcoholic steatohepatitis (NCT04531150) (Inversago Pharma) TM38837 in healthy subjects [104] (7TM Pharma) GFB-024 in diabetic nephropathy (Goldfinch Bio, NCT04880291)	AZD1940 in capsaicin-induced pain [124] and post-operative pain [125] ART27.13 (previously AZD1940) in Cancer anorexia (EudraCT NUMBER:2020-000464-27) (Artelo Biosciences)	URB937 is in the early stages of clinical development (Exxel Pharma)

### 3.2. Peripherally Restricted CB_1_ Agonists

After the discovery of the CB_1_ receptors and their important role in pain modulation, the first significant drug discovery program for peripherally restricted CB_1_ agonists was analgesics. The concept was to utilize the analgesic effects of CB_1_ activation without the CNS side effects, and extensive preclinical studies have demonstrated the analgesic effects of these compounds across various models of pain [126]. However, a lack of efficacy in clinical studies [124,125] meant the pharmaceutical development of these medicines was terminated. However, preclinical research with peripherally restricted CB_1_ agonists continues in cancer-related pain [109,110] and migraine [111]. Other indications that have been investigated with a peripherally restricted CB_1_ agonist included spasticity in multiple sclerosis [112], gastrointestinal motility issues [42,43], and anticipatory nausea [113], although none of these have been taken to clinic (see Table 2).

**Figure 2:**
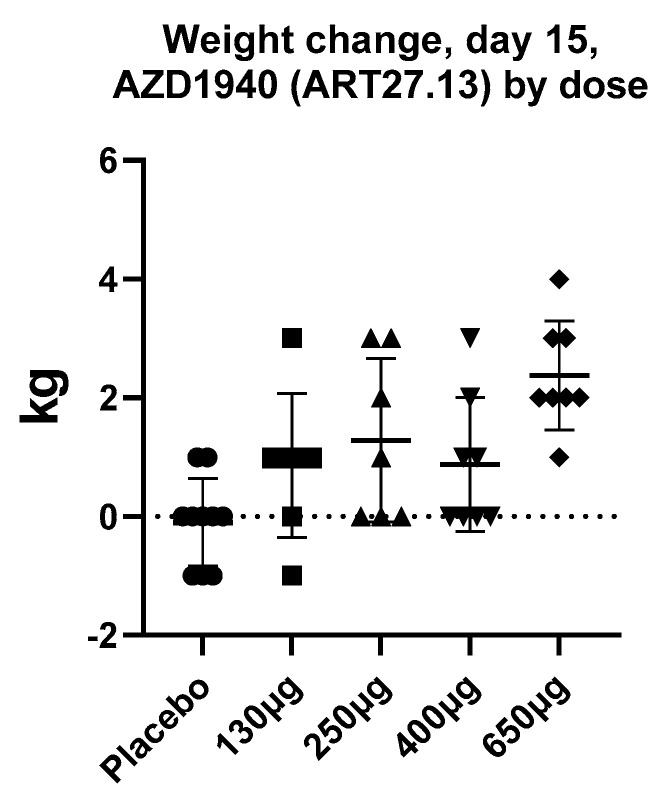
The mean increase in body weight (kg) after 15 days daily treatment with AZD1940 (ART27.13) in healthy volunteers in a dose-ascending study (ClinicalTrials.gov Identifier: NCT00689780, data on file). Data are a presented as a scatterplot with mean and SD.

By contrast to the large number of peripherally restricted CB_1_ antagonists in development for obesity and related metabolic disorders, far less work has been carried out to potential exploit CB_1_ activation in the periphery to promote weight gain. Although appetite stimulants such as the progesterone megestrol acetate, and the steroid dexamethasone, have been used for treatment of anorexia associated with cancer, no drugs have been approved for this indication in the United States or Europe, with the exception of dronabinol, which is approved for HIV/AIDS-induced anorexia only. Thus, the development of novel pharmaceutical strategies to stimulate appetite in chronic states of anorexia (such as cancer, chronic kidney disease, and heart failure) is still a significant unmet need. ART27.13 is a CB_1_/CB_2_ receptor agonist with reduced brain penetration originally developed by AstraZeneca for analgesia, now being developed by Artelo Biosciences. In a multiple-dose ascending study, a dose-dependent increase in body weight was observed (see Figure 2, ClinicalTrials.gov Identifier: NCT00689780, data on file) that was not explained by fluid retention; it was likely due to increased appetite and food intake. The clinical potential of ART27.13 to increase appetite leading to weight gain in patients with cancer anorexia is being trialed in a Phase 1b/2a study (EudraCT NUMBER:2020-000464-27).

### 3.3. Peripherally Restricted Fatty Acid Amide Hydrolase (FAAH) Inhibitors

Indirect activation of peripheral cannabinoid receptors can also be achieved through peripherally restricted fatty acid amide hydrolase (FAAH) inhibitors, which increase endocannabinoid tone and promote activation of cannabinoid receptors. Such compounds have been shown in preclinical research models to be analgesic in many models, including neuropathic pain [115], diabetic neuropathy [119], chemotherapy (paclitaxel)-induced pain [116], inflammatory pain [115,117,119], visceral pain [115], and migraine and medication overuse headache [120,121] (see Table 2). Peripherally restricted FAAH inhibitors also reduce anticipatory nausea [113], protect against non-steroidal anti-inflammatory agent-induced gastric lesions [118], and reduce hyperactivity in the rat bladder induced by PGE prostaglandin E2 [123] and in an LPS model of cystitis [122].

The peripherally restricted FAAH inhibitor URB937 is in development by ExxelPharma for chronic neuropathic pain; although human clinical studies have not yet begun (https://exxelpharma.com/pipeline/overview/ (accessed on 10 September 2021)), the use of this alternative strategy to activate peripheral cannabinoid receptors looks promising. 

## 4. Conclusions

Drug discovery efforts to develop CB_1_ agonists and antagonists were hampered by CNS-mediated side effects of these drugs. Second- and third-generation compounds in this area have tried to circumvent these adverse effects by selectively activating the CB_1_ receptor expressed in the peripheral nervous system and major organ systems of the body. Preclinical investigation supports the importance of the CB_1_ receptor throughout the gastrointestinal tract, adipose tissue, liver, pancreas, and skeletal muscle, as well as mediating humoral and afferent satiety signals to the brain. Preclinical efficacy data support the therapeutic utility of peripherally restricted CB_1_ agonists in pain management, and antagonists in obesity, metabolic syndrome, and liver diseases. Preclinical data also support indirect activation of peripheral CB_1_ receptors through peripherally-restricted FAAH inhibitors in pain management and bladder conditions. Translation of these findings into the clinical arena is emerging, with several pharmaceutical companies developing novel medicines in early phase 1 and 2 trials in weight gain in cancer anorexia (agonist: ART27.13), and in metabolic conditions (antagonists: INV-101, TM38837, and GFB-024), which, if successful, could result in novel, rationally designed synthetic cannabinoid medicines that demonstrate the appropriate benefit–risk profile to allow mainstream use in the modulation of weight by targeting CB_1_.

## Data Availability

Not applicable.
